# Association between adult adhd and generalised joint hypermobility, with and without systemic manifestations: A case-control study

**DOI:** 10.1192/j.eurpsy.2021.263

**Published:** 2021-08-13

**Authors:** M. Glans, S. Bejerot, M. Humble, M. Elwin, N. Thelin

**Affiliations:** 1 Faculty Of Medicine And Health, Örebro University, Örebro, Sweden; 2 Division Of Psychiatry, Linköping University Hospital, Linköping, Sweden

**Keywords:** comorbidity, Hypermobility, ADHD, biomarkers

## Abstract

**Introduction:**

There is growing evidence that generalised joint hypermobility (GJH) is associated with several psychiatric conditions. There are no previous studies on adult ADHD.

**Objectives:**

To evaluate, in a large Swedish sample, if generalised joint hypermobility and adult ADHD are associated.

**Methods:**

431 adults with ADHD and 417 controls were included. GJH was assessed by the Beighton Score, a physical examination, and the 5PQ, a self-report screening tool. Exploratively, reported musculoskeletal symptoms and abnormal skin manifestations suggestive of symptomatic GJH (e.g. Ehlers-Danlos syndrome), were assessed to differentiate this group from the general GJH group. Logistic regressions determined the influence of an ADHD diagnosis and known covariates (age, sex and ethnicity) on GJH and symptomatic GJH respectively.

**Results:**

ADHD was associated to GJH, as defined by the Beighton Score and the 5PQ, with adjusted odds ratios of 4.65 (CI 95% 3.01-7.18, p<.005) and 1.86 (CI 95% 1.39-2.48, p<.005), respectively. Likewise, ADHD and symptomatic GJH were associated with adjusted odds ratios of 6.94 (CI 95% 4.05-11.89, p<.005) and 2.66 (CI 95% 1.94-3.66, p<.005).

**Conclusions:**

GJH and adult ADHD are associated conditions. Symptomatic GJH, defined as additional symptoms of pain and/or skin manifestations, has a considerably stronger link to adult ADHD than unspecific GJH has. GJH may represent a marker of an underlying systemic disorder with physical manifestations in connective tissue as well as behavioural manifestations including hyperactivity, impulsiveness and inattentiveness. Future studies should investigate if this represents a novel subtype of ADHD and if symptomatic GJH affects the ADHD management.

**Disclosure:**

No significant relationships.

